# Integrated analysis of senescence-associated genes in pancreatic ductal adenocarcinoma

**DOI:** 10.3389/fgene.2022.941389

**Published:** 2022-08-15

**Authors:** Zhi-gang Zhu, Lei Chen, Dong-liu Miao, Yiqi Jin, Qiong Wu

**Affiliations:** Department of Intervention and Vascular Surgery, Suzhou Municipal Hospital, Affiliated Suzhou Hospital of Nanjing Medical University, Suzhou Cancer Medical Center, Suzhou, China

**Keywords:** senescence, pancreatic ductal adenocarcinoma, prognostic signature, tumor microenvironment, immunotherapy

## Abstract

**Background:** Cellular senescence plays a critical role in the occurrence and development, and immune modulation of cancer. This research primarily investigated the role of senescence-associated genes (SAGs) in the survival and tumor microenvironment of pancreatic ductal adenocarcinoma (PDAC).

**Methods:** From the Cancer Genome Atlas (TCGA) and International Cancer Genome Consortium (ICGC) database, the gene expression profiles and clinical data of PDAC samples were downloaded. SAGs in the TCGA cohort were used to build a novel prognostic model and validated in the ICGC cohort. The relationship of signature with the immune landscape, tumor mutational burden (TMB), as well as the sensitivity of different therapies, was explored. Moreover, a nomogram was developed to predict the overall survival of PDAC patients.

**Results:** A prognostic signature was constructed on basis of three SAGs, and patients in the low-risk score group had a longer survival time. The accuracy of the signature to distinguish different score groups was confirmed through principal component analysis (PCA) and the Receiver operator curves curve. The mRNA expression of the three signature genes was also verified in normal pancreatic and PDAC cell lines by RT-qPCR. The signature could independently predict the prognosis of PDAC patients and had broad applicability. Meanwhile, the nomogram predicted that 1- and 3-years survival rates were in good agreement with the observed overall survival rates. Low-risk patients had lower tumor mutational burden, and low-TMB patients had a better prognosis. Low- and high-risk patients exhibit distinct immune cell infiltration and immune checkpoint changes. By further analyzing the risk score, patients in the low-risk group were more responsive to immunotherapy and a variety of commonly used chemotherapeutic drugs.

**Conclusion:** The prognostic signature can well predict the prognosis and assess the possibility of immunotherapy in personalized PDAC treatment.

## Introduction

Pancreatic ductal adenocarcinoma (PDAC) is a highly malignant gastrointestinal tumor and is projected to become the second-leading cause of cancer-related mortality by 2030 (Mizrahi et al., 2020; Siegel et al., 2022). Due to the influence of pancreatic anatomy and its biological characteristics, PDAC is prone to invade surrounding tissues and organs and develop distant metastasis in the early stage. In addition, there are no obvious and specific symptoms and signs in the early stage. Most patients are diagnosed in the advanced stage and have a very poor prognosis (Mizrahi et al., 2020). Surgical resection is still the only cure for PDAC, but only 15%–20% of PDAC patients currently have the opportunity to seek curative treatment through surgery, and most patients rely mainly on adjuvant systemic chemotherapy to improve their prognosis (Mizrahi et al., 2020). However, even a combination of surgery, systemic chemotherapy, and radiotherapy benefits only a small proportion of patients with PDAC (Narayanan et al., 2021). Therefore, it is particularly important to find new and feasible molecular pathological markers and therapeutic targets for early identification and appropriate management.

Intratumor heterogeneity in tumors results from distinct genetic aberrations, immune microenvironments, metastatic capacity, and senescence (Kim et al., 2017). Among them, senescence is an important regulatory mechanism. Senescence refers to a cellular state characterized by stable cell cycle arrest in response to various stresses (Hernandez-Segura et al., 2018). Senescent cells have the dual role of promoting and alleviating cancer. In response to various stresses, they enter persistent cell cycle arrest to inhibit proliferation, thereby inhibiting tumorigenesis of precancerous cells. Senescent cells can exhibit a variety of features, including elevated reactive oxygen species (ROS), cell cycle regulators, and a senescence-associated secretory phenotype (SASP) (Prieto and Baker, 2019). However, SASPs produced by senescent cells can also disrupt the surrounding environment for tumor growth, recurrence, and metastasis (Ruhland et al., 2016; Demaria et al., 2017). SASP promotes senescence of immune cells, including macrophages, in an autocrine manner, thereby evading tumor cell surveillance and clearance of senescent cells (Behmoaras and Gil, 2021). In addition, the accumulation of senescent cells will promote the release of SASP factors and promote the growth of tumor cells (Prieto and Baker, 2019). These observations suggest that tumors are heterogeneous in their senescence status. Therefore, personalized treatment is the hot spot for the treatment of PDAC and it is also an urgent task at current.

In recent years, gene expression signatures based on SAGs have been reported to predict the prognosis of various cancers, including lung adenocarcinoma, glioma, and colorectal cancer (Lin et al., 2021; Luo et al., 2021; Yue et al., 2021). However, PDAC patients lack such a prognostic signature associated with senescence. In present research, we established a novel prognostic model for patients based on SAGs to predict OS in PDAC patients and guide individualized treatment.

## Materials and methods

### Data source

The RNA transcriptome dataset and related clinical information of 178 PDAC patients and 4 normal samples were downloaded from The Cancer Genome Atlas (TCGA, https://portal.gdc.cancer.gov/). Due to the lack of normal pancreatic tissue data in the TCGA database, we downloaded Genotype-Tissue Expression (GTEx, https://www.gtexportal.org/home/datasets) data from 167 normal pancreatic samples to identify the DEGs between normal and tumor tissues. TCGA data filtering criteria are as follows: 1. Complete prognostic information; 2. Survival time is more than 1 month; 3. Remove duplicate samples and normal samples. Following then, a total of 170 PDAC patients were included in the studies. In addition to this, the gene expression profiles and clinical information of 82 PDAC samples were downloaded from the International Cancer Genomics Consortium (ICGC, https://dcc.icgc.org/). The data of 170 PDAC patients in TCGA were used as the training set to build a prognostic model, and the ICGC dataset (82 patients) was used as an external validation cohort. In addition, 279 senescence-associated genes were collected from the CellAge database (https://genomics.senescence.info/cells/signatures.php).

### Development of a senescence-associated signature and a nomogram

To identify the prognostic SAGs, we performed univariate analysis in the training set. Then, LASSO regression analysis was applied to narrowing down candidate genes using the R package “glmnet”. Subsequently, multivariate Cox regression analysis was performed to build a signature. The following formula was employed to calculate the risk scores of PDAC samples:

$$\mathrm{Risk}\;\mathrm{score}=\sum_{i=1}^{n}Coef\left(xi\right)\times Exp\left(xi\right)$$

“Coef” represents the non-zero regression coefficient and “Exp” represents the expression level of genes in the signature. The samples were equally classified into testing and training sets. Patients were categorized into high- and low-risk score groups according to the median value of risk scores. Kaplan-Meier analysis was performed in the high and low-risk groups with the “survival” and “survminer” packages.

Receiver operator curves (ROC) were drawn with “timeROC” package to assess the signature’s accuracy in predicting survival time. We also verified whether the signature could distinguish between different risk score groups based on PCA analysis. The risk state diagram and survival state diagram were plotted. Univariate and multivariate regression analyses were performed to confirm if the signature could be considered an independent predictor signature. The nomogram was constructed with “rms” package combined with clinicopathological variables. The same method was used to calculate the ICGC cohort’s risk score and perform risk grouping to verify the predictive power of the signature.

### Assessment of immune cell infiltration and the tumor microenvironment in different risk groups

To explore the differences in the tumor immune microenvironment between the high- and the low-risk group, we calculated the proportion of infiltrating immune cells in the PDAC samples according to the CIBERSORT algorithm. The ESTIMATE algorithm was employed to investigate the stromal, immune and ESTIMATE score between the two groups.

### Investigation of differences in immunotherapy and chemotherapeutic efficacy

The expression of immune checkpoints in high- and low-risk groups was analyzed, and Tumour Immune Dysfunction and Exclusion (TIDE) algorithm was employed to estimate the response to immunotherapy (Jiang et al., 2018). The response of each sample to anti-CTLA4 and anti-PD-1/PD-L1 immunotherapy was assessed using TIDE algorithm based on the gene expression profiles. In addition, the IC50 of chemotherapeutic drugs in PDAC was calculated with the “pRRophetic” package.

### Relationship between risk score and tumor mutational burden

We calculated the mutation frequency and the number of variants in each sample and assessed the mutation status of the genes in the high- and low-risk subgroups. The difference between somatic mutation and TMB was compared between different risk groups. Patients with PDAC were classified into the low-TMB and the high-TMB group. Based on TMB survival analysis to explore the survival differences in different TMB groups. Next, risk score and tumor mutation load were combined to perform survival analysis to determine if there are differences in patients between different groups.

### Cell culture and qRT-PCR

The RNA transcriptome data of PDAC patients and normal samples from TCGA and GTEx databases were used to investigate the expression level of signature genes in PDAC tissues and normal pancreatic tissues. In addition, the normal pancreatic cell line hTERT-HPNE and two human PDAC cell lines (PANC-1, SW 1990) were purchased from the American Type Culture Collection (ATCC) and the Type Culture Collection of the Chinese Academy of Sciences (Shanghai, China). Cell lines were cultured using RPMI-1640 or DMEM (HyClone, Logan, UT, United States ) containing 10% fetal bovine serum (FBS, Gibco, United States ) and 1% penicillin-streptomycin (P/S) (Gibco, United States ). Cell lines were grown in a sterile and humidified cell culture incubator at 37°C and 5% CO_2_.

Total RNAs from PDAC cell lines were isolated using TRIzol reagent (Invitrogen, United States ), and cDNA was generated with Prime ScriptTM RT Master Mix (Takara, Japan). This was followed by the performance of RT-qPCR with TB Green^®^ Premix Ex Taq™ II (Takara, Japan) using Glyceraldehyde-3-phosphate dehydrogenase (GAPDH) as an internal control. The 2^−ΔΔCt^ method was performed to calculate the mRNA relative expression of genes.

## Results

### Establishment of a senescence-associated prognostic signature

**FIGURE 1:**
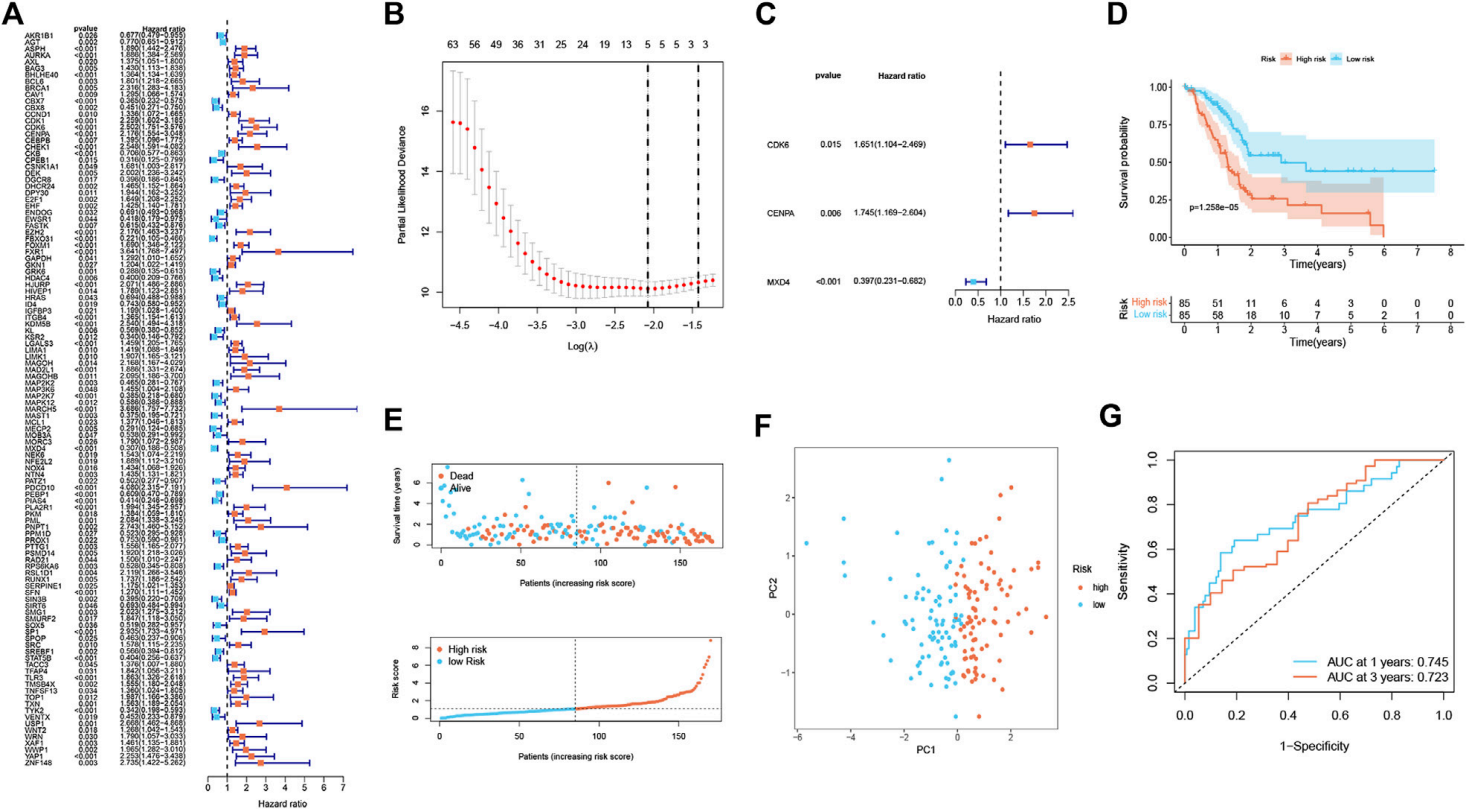
Establishment of a senescence-associated prognostic signature in the training cohort (*n* = 170). **(A)** The prognostic SAGs were selected by univariate Cox regression analysis. **(B)** 10-fold cross-validation in the LASSO signature. **(C)** The presentation of three independent prognosis genes in multivariate Cox regression analysis. **(D)** Survival analysis between low- and high-risk subgroups. **(E)** Survival status and risk score distribution. **(F)** PCA analysis displayed an obvious difference in transcriptomes between two risk groups. **(G)** ROC curve. The accuracy of the signature in predicting 1- and 3-years survival of PDAC patients was evaluated.

**FIGURE 2:**
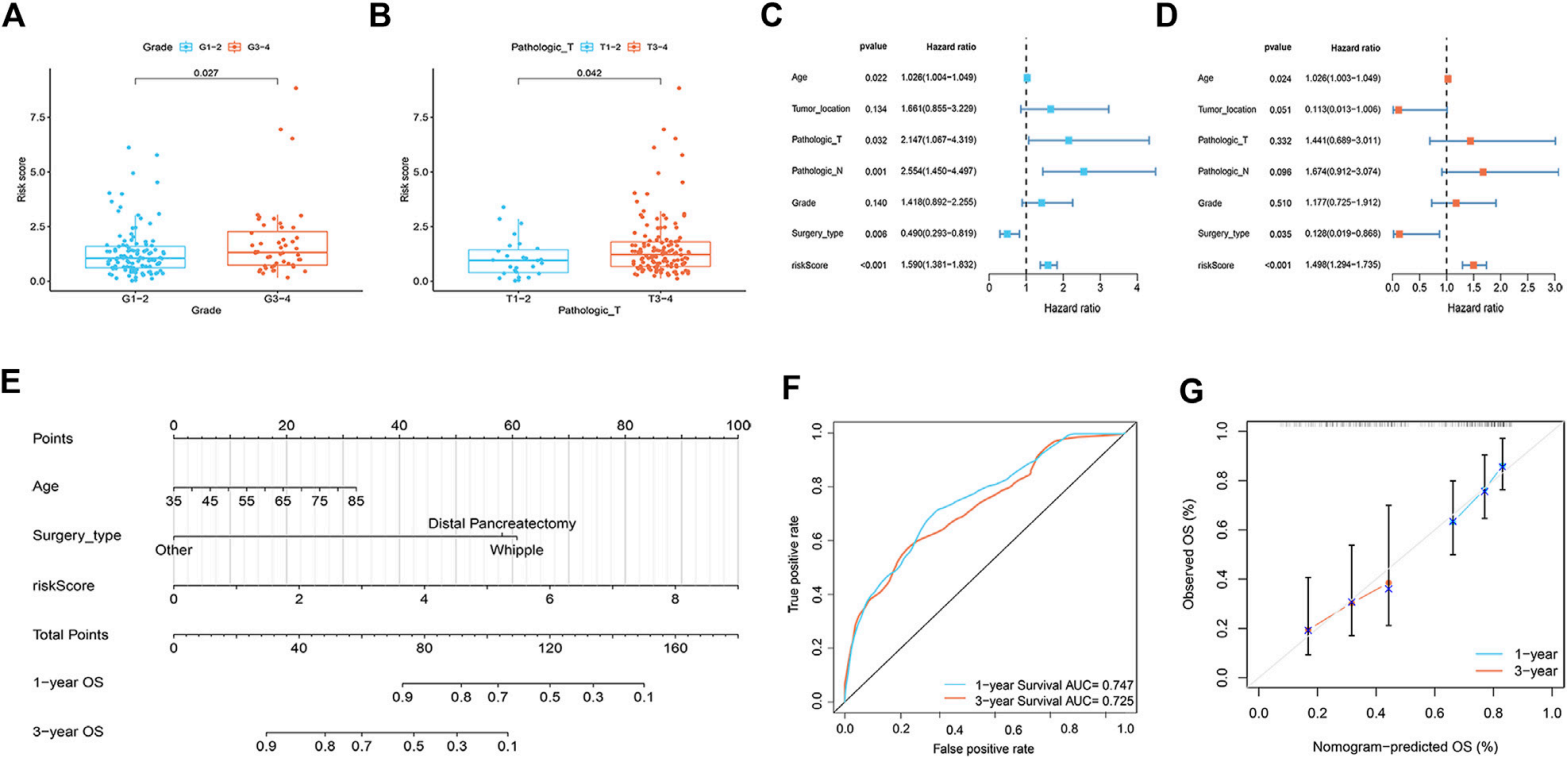
Correlation analysis and nomogram construction (*n* = 161). **(A)** The relationship between risk score and tumor grade. **(B)** The relationship between risk score and pathologic T stage. **(C,D)** Forest plots of univariate **(C)** and multivariate **(D)** Cox regression analysis, including risk scores and clinicopathological variables. **(E)** The nomogram of combined risk score and clinical parameters. **(F)** ROC curve was used to evaluate the accuracy of nomogram. **(G)** Calibration plots for predicting OS rates at one and 3 years.

A total of 112 prognostic SAGs were identified by univariate Cox analysis (Figure 1A) and were subject to LASSO Cox regression analysis to avoid overfitting, and 5 SAGs were chosen as the appropriate candidates for constructing a risk signature (Figure 1B). Subsequently, multivariate Cox regression analysis obtained 3 genes (CDK6, CENPA, and MXD4) to build a prognostic signature (Figure 1C). The risk score under the prognostic model was next calculated according to the gene expressions and optimal coefficients. The risk formula was 0.5011*expression (CDK6) + 0.5567* expression (CENPA)—0.9247* expression (MXD4). Patients were divided into high- and low-risk groups according to the median value of the training group. Compared with the low-risk group, the prognosis of patients in the high-risk group was poor (Figure 1D). PDAC patients were ranked based on the risk scores, and their distribution was shown in Figure 1E. The scatter plot showed that the survival status of PDAC patients was related to the risk score, and as the risk score increased, the mortality of the patients increased (Figure 1E). PCA analysis revealed that there was a clear division between low- and high-risk score groups (Figure 1F). The area under the curve (AUC) of ROC curves showed that the risk score had good prediction accuracy (Figure 1G). Furthermore, we compared our signature with 9 previously developed models of pancreatic cancer. Except for the prognostic signature constructed by Cai et al., the AUC value of our signature is higher than that of most prognostic models, indicating that our model has better predictive ability (Supplementary Figure S1). To explore the clinical value of the signature, we further investigated the association between the risk score and each clinical characteristic. The results demonstrated that risk score was linked to grade (*p* = 0.027) and pathologic T stage (*p* = 0.042; Figures 2A,B). To confirm the prognostic signature whether can be used as an independent prognostic indicator for PDAC patients or not, Cox regression analysis was performed and two forest plots were drawn. The result showed that risk score, age, and surgery type were independent prognostic indicators (Figures 2C,D). We also built a nomogram based on clinicopathological features and risk scores (Figure 2E), which could predict PDAC patients’ 1- and 3-years survival rates. At the same time, one PDAC patient was randomly selected for scoring, the results of which were shown in Figure 2E. The ROC curve revealed the high accuracy of the nomogram for 1-year (AUC = 0.747) and 3 -year (AUC = 0.725) survival rates (Figure 2F). Compared to the TNM stage, our model predicted a more accurate prognosis at the 1- and 3-years time points (Supplementary Figure S2). Also, we confirmed the good agreement between nomogram predictions of 1- and 3-years survival and observed OS rate using calibration curves (Figure 2G).

**FIGURE 3:**
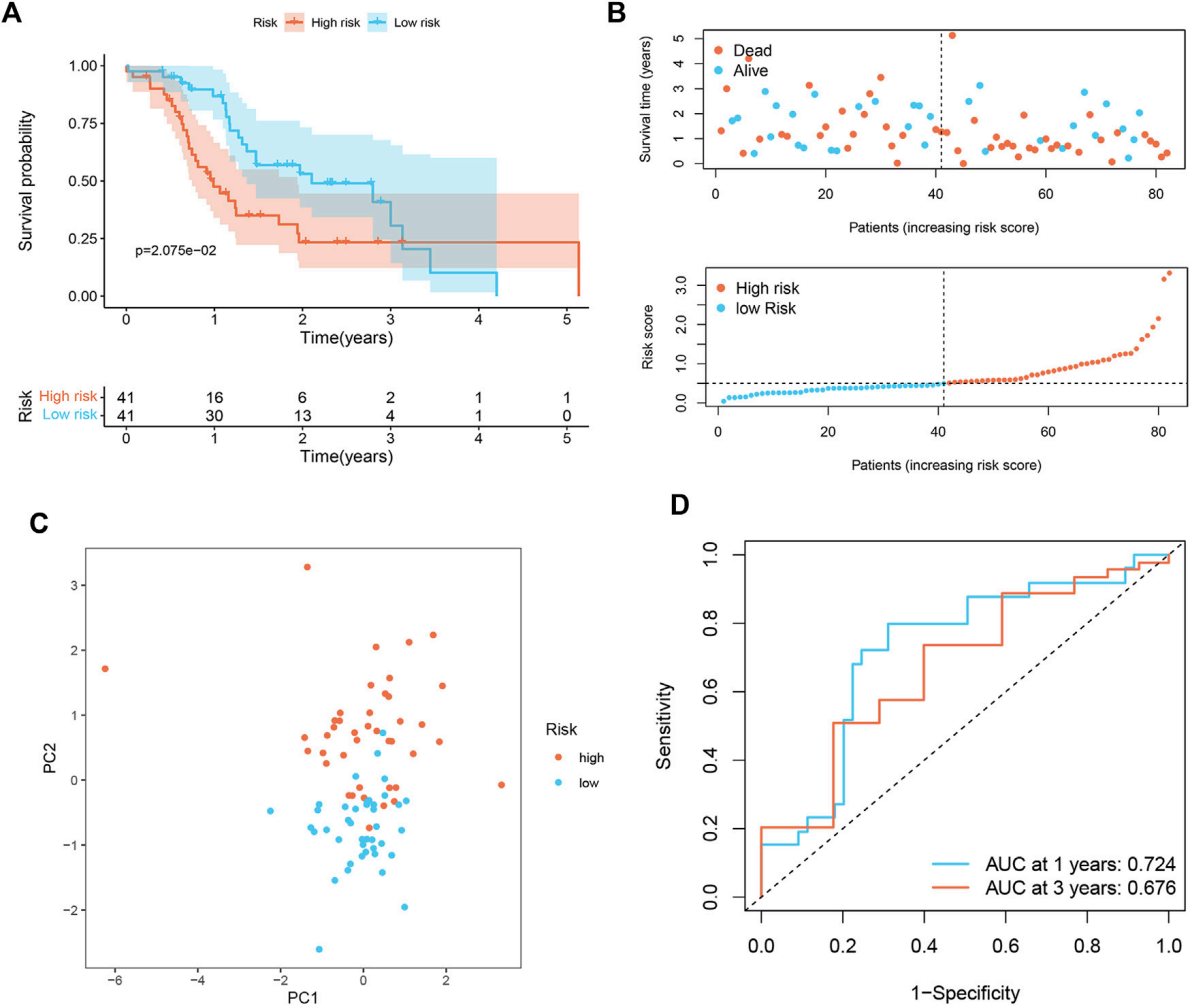
Validation of senescence-associated signature in ICGC cohort (*n* = 82). **(A)** Kaplan-Meier analysis of OS among different subgroups. **(B)** Survival status among two risk subgroups and risk score distribution. **(C)** PCA analysis distinguished between high- and low-risk score groups. **(D)** ROC curve evaluates the prediction accuracy of risk score.

To prove the applicability of the risk score, we conducted external validation. In the ICGC set, the signature still had good predictive performance. The survival rate of PDAC patients with high-risk was significantly lower than that of patients with low-risk (Figure 3A). The distribution of risk score, survival status, and OS time of the two groups is shown in Figure 3B. PCA demonstrated overt separation of both subgroups (Figure 3C). AUC showed that the risk score had high prediction accuracy (Figure 3D).

### Tumor microenvironment characteristics in the different risk groups

**FIGURE 4:**
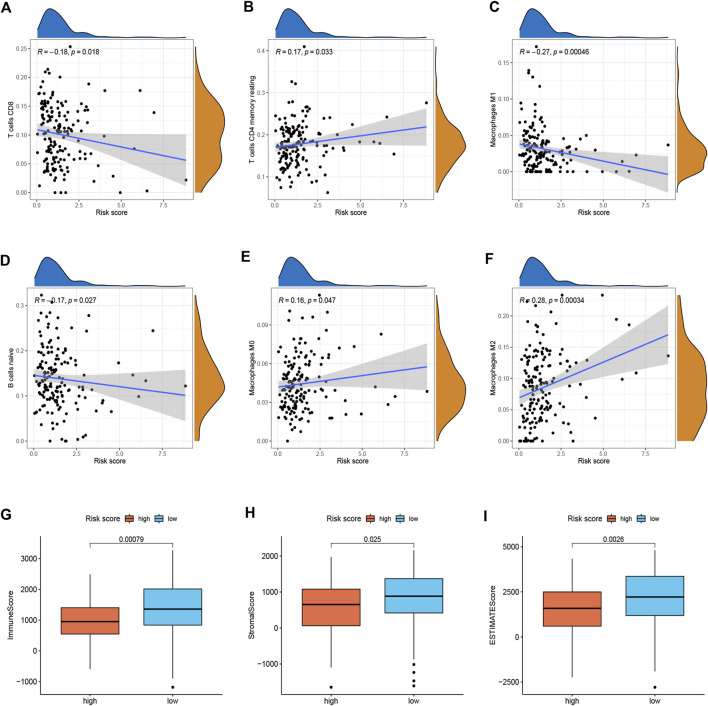
Immune landscape between two risk subgroups (*n* = 170). **(A–F)** Immune cell infiltration among different subgroups. **(G–I)** Immune, stromal, and ESTIMATE scores among different subgroups.

To better investigate the relationship between risk score and immune characteristics, CIBERSORT algorithms were used to calculate the enrichment scores of various immune cells. As is shown in Figures 4A–F, the risk score was found to be negatively correlated with CD8^+^ T cells, resting memory CD4^+^ T cells, M1 macrophages, and naive B cells and positively correlated with M0 macrophages and M2 macrophages. We further explored differences in immune infiltrating cells between low- and high-risk groups. The results showed that B cells naive, CD8^+^ T cells, Monocytes, Macrophages M0, Macrophages M1, and Macrophages M2 were significantly different between the low-risk group and the high-risk group. B cells naive, CD8^+^ T cells, Monocytes, and Macrophages M1 cells infiltrated more in the PDAC samples from low-risk score group. Meanwhile, the infiltrations of Macrophages M0 and Macrophages M2 cells were higher in the high-risk score group (Supplementary Figure S3). In addition, we found that the immune, stromal, and ESTIMATE score of high-risk patients were significantly lower than those of low-risk patients (Figures 4G–I).

### Immune therapeutic evaluation of and chemotherapeutic drug selection

**FIGURE 5:**
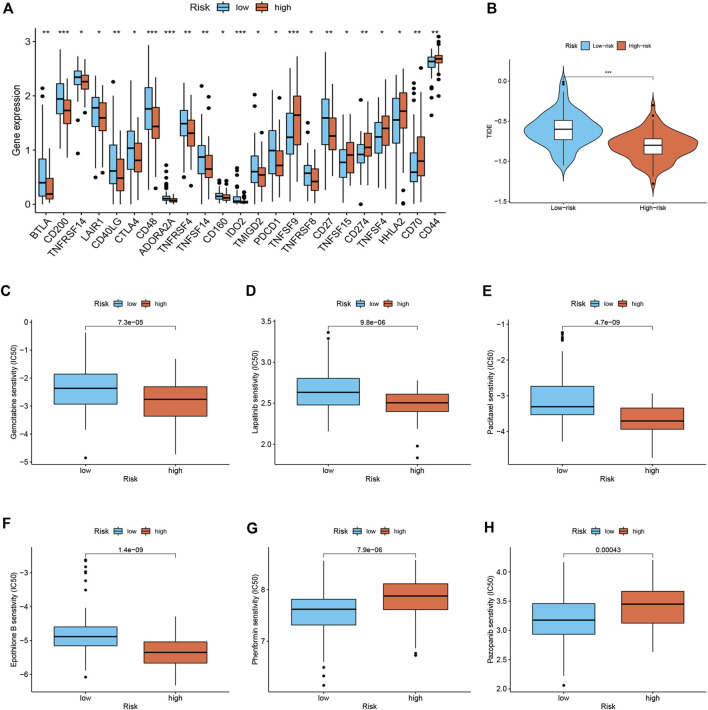
Immune therapeutic evaluation of and chemotherapeutic drug selection (*n* = 170). **(A)** Differences in immune checkpoint among different risk subgroups. **(B)** Comparison of TIDE score between low- and high-risk groups. **(C–H)** Comparison of potential therapeutic drug susceptibility of different risk subgroups assessed by IC50.

Considering the relevance of ICIs in treating PDAC, we investigated the potential role of the signature in assessing the immunotherapy efficacy of ICIs in PDAC patients by analyzing the association between the signature and prevalent ICIs targets. PD-L1 (CD274) was highly expressed in high-risk score group, while the CTLA4 and PD1 (PDCD1) were highly expressed in low-risk score group (Figure 5A). Furthermore, we applied the TIDE algorithms to evaluate the effectiveness of the signatures in forecasting ICIs responsiveness in PDAC. Patients with high-risk scores had a lower TIDE score compared with those with low-risk scores (Figure 5B). Taken together, patients with high-risk scores can predict the benefit of PDAC immunotherapy. Distinct PDAC subgroups should guide clinical treatment. To identify PDAC patients with drug-sensitive, the sensitivity of the different risk score groups to the drugs was further investigated. Gemcitabine, Lapatinib, Paclitaxel, and Epothilone B had lower IC50 in the high-risk group (Figures 5C–F), and Phenformin and Pazopanib had higher IC50 in the high-risk group (Figures 5G,H).

### Mutation landscape of different risk score groups

**FIGURE 6:**
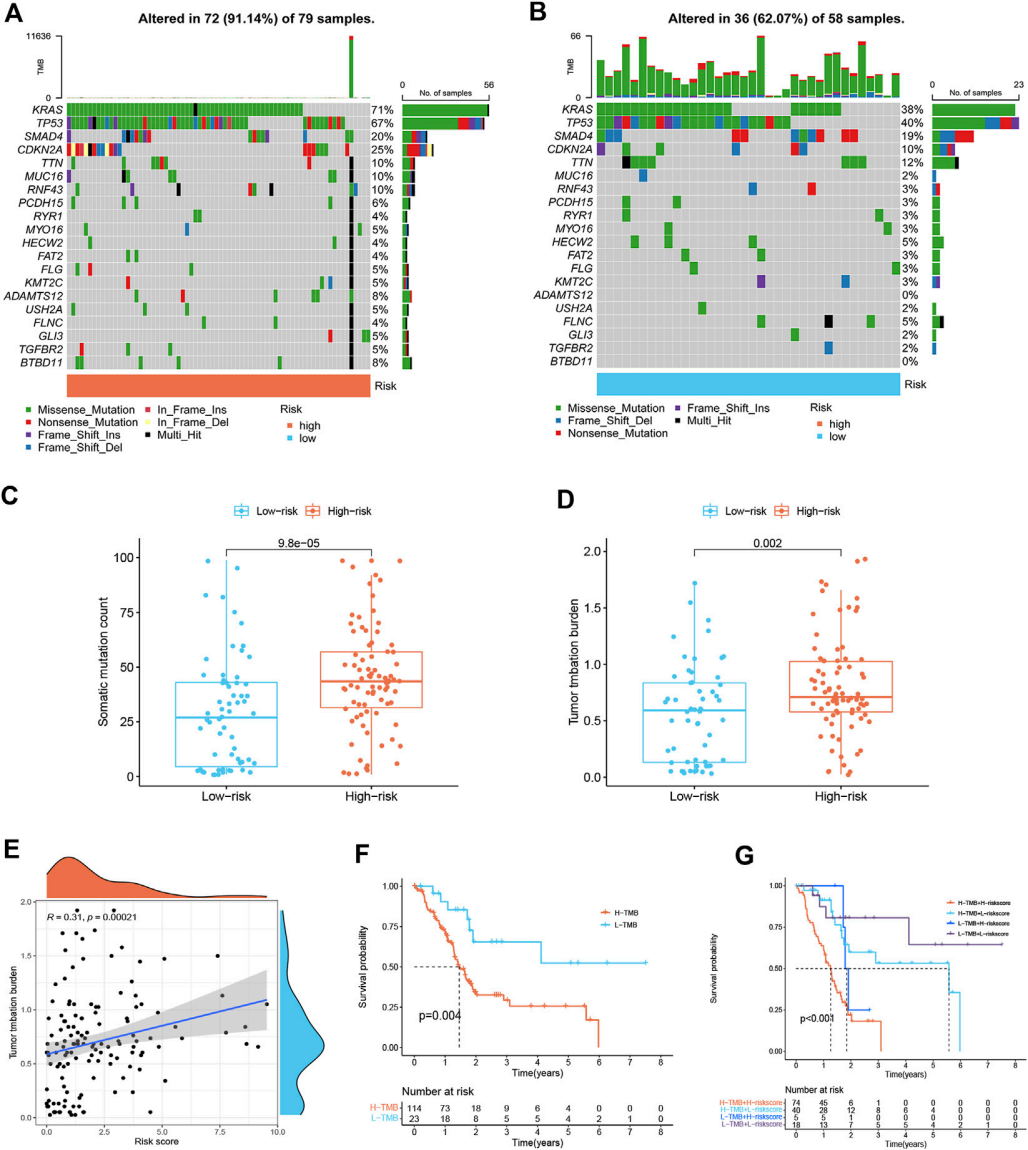
Mutation landscape of different risk score subgroups (*n* = 137). **(A,B)** Waterfall plots show higher mutation frequency genes in high- **(A)** and low-risk groups **(B)**. **(C)** Boxplot for differences in somatic mutation count between two risk subgroups. **(D)** Boxplot for differences in TMB between two risk subgroups. **(E)** Correlation analysis between TMB and risk score. **(F)** Survival curves of PDAC patients in H-TMB and L-TMB groups. **(G)** Survival curves. The risk score and TMB were combined to perform survival analysis.

Differences in gene mutation frequencies were analyzed in different risk score groups. There was a marked difference in mutation frequency between the two groups. The mutation rate in the high-risk group was 91.14% (Figure 6A), whereas 62.07% was in the low-risk group (Figure 6B). From the waterfall charts, it can be seen that the mutated genes in two risk subgroups were mainly KRAS, CDKN2A, SMAD4, and TP53. Somatic mutation count and TMB were higher in the high-risk group (Figures 6C,D), and TMB was positively associated with risk score (Figure 6E), suggesting that immunotherapy was more effective in high-risk patients. In addition, we found that low TMB patients have a better prognosis (Figure 6F). Stratified survival analysis demonstrated that the effectiveness of the risk score in predicting survival in PDAC was not influenced by TMB (Figure 6G). This indicated that the risk score could be regarded as an independent predictor.

### Validation of the expression of signature genes in pancreatic ductal adenocarcinoma

We analyzed the expression level of three signature genes in PDAC tissues and normal pancreatic tissues. Compared with normal pancreatic tissues, CDK6 and CENPA were up-regulated in PDAC tissues, while MXD4 was down-regulated in PDAC tissues (Supplementary Figure S4A). To further validate the clinical practicability of the signature, we compared the expression levels of three signature genes in two PDAC cell lines (PANC-1, SW 1990) and a normal pancreatic cell line (hTERT-HPNE) by qRT-PCR. As shown in Supplementary Figures S4B–D, the expression of CDK6 and CENPA in both PANC-1 and SW1990 cell lines was higher than that in the hTERT-HPNE cell line, while the expression of MXD4 in both PANC-1 and SW1990 cell lines was lower than that in the hTERT-HPNE cell line.

## Discussion

PDAC is a disease with a dire prognosis and one of the few cancers with a rising incidence (Gheorghe et al., 2020). Growing evidence suggests that its malignant behavior is largely influenced by the associated strong immunosuppression, relatively low mutational burden, and desmoplastic microenvironment (Cortesi et al., 2021). During the early stages of tumorigenesis, cancer cells must shed the effects of cellular senescence, which slows proliferation and promotes immune-mediated clearance of precancerous cells. However, recent evidence has revealed that senescent cells promote the senescence of macrophages by secreting SASP factors. Subsequently, senescence-associated macrophages may affect other immune cells to escape tumor cell surveillance and senescent cell clearance (Prieto and Baker, 2019). SASP is a double-edged sword that recruits and activates immune cells as well as neighboring cells, resulting in pro-tumor and anti-tumor effects (Toso et al., 2015). Therefore, modeling PDAC has important implications for deciphering whether molecular determinants of senescence remodel the TME and whether this modification has an impact on prognosis and immunotherapy response in patients with PDAC.

In our study, TCGA and ICGC datasets serve as the training and validation cohorts, respectively. We refined LASSO and Cox regression analyses on the extracted prognostic genes in the training cohort to build a prognostic signature. The signature contained three SAGs: CDK6, CENPA, and MXD4. Beyond that, the validation of expression levels of three genes in the signature by RT-qPCR further demonstrated the feasibility of the signature in clinical application. CDK6 is a key component of the cell cycle machinery, driving the G1 to S phase transition of the cell cycle by phosphorylating and inactivating retinoblastoma protein (RB) (Gao et al., 2020). Dysregulation of CDK6 activity affects various aspects of cancer cell proliferation, senescence, migration, apoptosis, and angiogenesis (Nebenfuehr et al., 2020). Dual CDK4/6 inhibitors have achieved great success in the treatment of hormone-receptor-positive breast cancer and have shown promising results in several solid tumors and hematological malignancies (Fassl et al., 2022). Rencuzogulları et al. (2020) revealed that Palbociclib, a selective CDK4/6 inhibitor, restricted cell survival and epithelial-mesenchymal transition (EMT) in PANC-1 and MIAPACA-2 pancreatic cancer cells. Ruscetti et al. (2020) indicated that a combination of CDK4/6 inhibitors and MEK could inhibit PDAC proliferation by inducing RB protein-mediated senescence. Senescent cells produce SASP to promote tumor vascularization, which in turn enhances drug delivery and efficacy. Furthermore, SASP-mediated endothelial activation stimulates CD8^+^ T cell recruitment into otherwise immunologically “cold” tumors, thereby sensitizing tumors to PD-1 checkpoint blockade (Ruscetti et al., 2020). CENPA has been reported to be an oncogene in various malignancies, including PDAC (Furukawa et al., 2006; Zhang et al., 2020; Han et al., 2021; Wang et al., 2021). Wang et al. (2021) revealed that CENPA overexpression promotes the proliferation and metastasis of clear cell renal cell carcinoma by activating the Wnt/β-catenin signaling pathway. MXD4, also known as MAD4, is the most abundant in the human brain and overexpression of MAD4 in human fibroblasts induces replicative senescence (Marcotte et al., 2003). Yang et al. (2012) observed that MAD4 exhibits stable steady-state expression in glioblastoma cell lines, and its proteins are involved in regulating c-Myc and E2F transcription factors and inducing cellular replicative senescence.

PDAC patients were categorized into two risk score groups, with high-risk scores being associated with poor prognosis. Interestingly, mortality of PDAC increases with increasing risk scores. The accuracy of the signature was demonstrated by using ROC and PCA analysis. In addition, the signature was externally validated in the ICGC dataset and still had a good predictive performance. Most importantly, we found that the risk score can be considered an independent predictor of PDAC prognosis. To better predict the prognosis of patients, we combined the surgery type and age to construct a nomogram, which improved the predictive performance of the risk score. Then, the relationship of risk scores with clinical features was further investigated. Risk scores were higher in patients with advanced T stage and tumor grade. To sum up, the model we constructed may be effective in determining prognosis, thereby facilitating the implementation and evaluation of the model in future clinical practice.

Highly heterogeneous cell subsets are characteristic of PDAC. This complex structure of cancer cells and stromal and immunosuppressive cells thus alters the efficacy of immunotherapy (Sarantis et al., 2020). The TME of PDAC is essentially immunosuppressive and includes regulatory tumor-associated macrophages (TAM), Treg cells, and myeloid-derived suppressor cells (MDSC). In this study, we analyzed the relationship between risk score and TME and found that the high-risk group presented a typical tumor immunosuppressive microenvironment. The risk score was positively associated with M2 macrophages and negatively associated with CD8^+^ T cells, resting memory CD4^+^ T cells, and M1 macrophages, indicating that the signature may contribute significantly to modulating immune cell infiltration. M2 macrophages have immunosuppressive properties that promote tumor progression (Lee et al., 2019). It has been reported that resident M2 macrophages were identified as highly proliferative and immunosuppressive, contributing to PDAC progression (Cortesi et al., 2021). Studies have shown that high M2 macrophage density was associated with worse OS in PDAC patients (Cortesi et al., 2021). Also, the StromalScore, ESTIMATEScore, and ImmuneScore of high-risk patients were significantly lower than those of low-risk patients.

Recently, immunotherapy has been known to play a significant role as a method to eradicate tumor cells based on ICIs among a subset of PDAC patients (Carpenter et al., 2021). In our study, a novel senescence-based signature was developed to investigate the relationship between ICIs and risk score as a predictor of immunotherapy response. PD-L1 (CD274) was highly expressed in high-risk score group, while the CTLA4 and PD1 (PDCD1) were highly expressed in low-risk score group, suggesting that high-risk patients may benefit more from anti-PD-L1 therapy. Furthermore, we found that high-risk patients had a lower TIDE score than those with the high-risk score. A lower TIDE score indicates a lower possibility of tumor immune evasion and may benefit from immunotherapy, which further explains the favorable prognosis of low-risk patients in our study. Evidence has indicated that patients with a higher TMB are more likely to benefit from immunotherapy owing to the existence of a greater number of neoantigens (Fusco et al., 2021). By TMB analysis, we found that TMB was significantly associated with prognostic signature and that the low-risk group had lower TMB and better prognosis. Stratified survival analysis revealed that risk score predicted the prognosis of PDAC patients completely independent of TMB. These results suggest that the signature can predict the benefit of PDACC immunotherapy. Finally, we identified potential chemotherapeutic drugs for PDAC patients. Patients with high-risk scores seem to be more responsive to Gemcitabine, Lapatinib, Paclitaxel, and Epothilone B, while low-risk patients were more sensitive to Phenformin and Pazopanib. The combination of chemotherapy and immunotherapy can provide precise and individualized therapy for patients with different risk scores.

## Conclusion

This study proposed a novel senescence-associated prognostic signature that will predict the prognosis of PDAC patients and provide a basis for the personalized treatment of PDAC patients.

## Data Availability

Publicly available datasets were analyzed in this study. This data can be found here: The data for this study can be found in TCGA (https://portal.gdc.cancer.gov/) and International Cancer Genomics Consortium (ICGC).

## Abbreviations

ICIs, Immune checkpoint inhibitors; OS, Overall survival; PCA, Principal component analysis; PDAC, Pancreatic ductal adenocarcinoma; SAGs, Senescence-associated genes; SASP, Senescence-associated secretory phenotype; TCGA, The Cancer Genome Atlas; TIDE, Tumour Immune Dysfunction and Exclusion; TMB, Tumor mutational burden; TME, tumor microenvironment.
