# Supplementary figures and images for: Integrated analysis of senescence-associated genes in pancreatic ductal adenocarcinoma
Source: Front Genet. 2022 Aug 15;13:941389. doi: 10.3389/fgene.2022.941389 (PMC9420911; doi:10.3389/fgene.2022.941389)

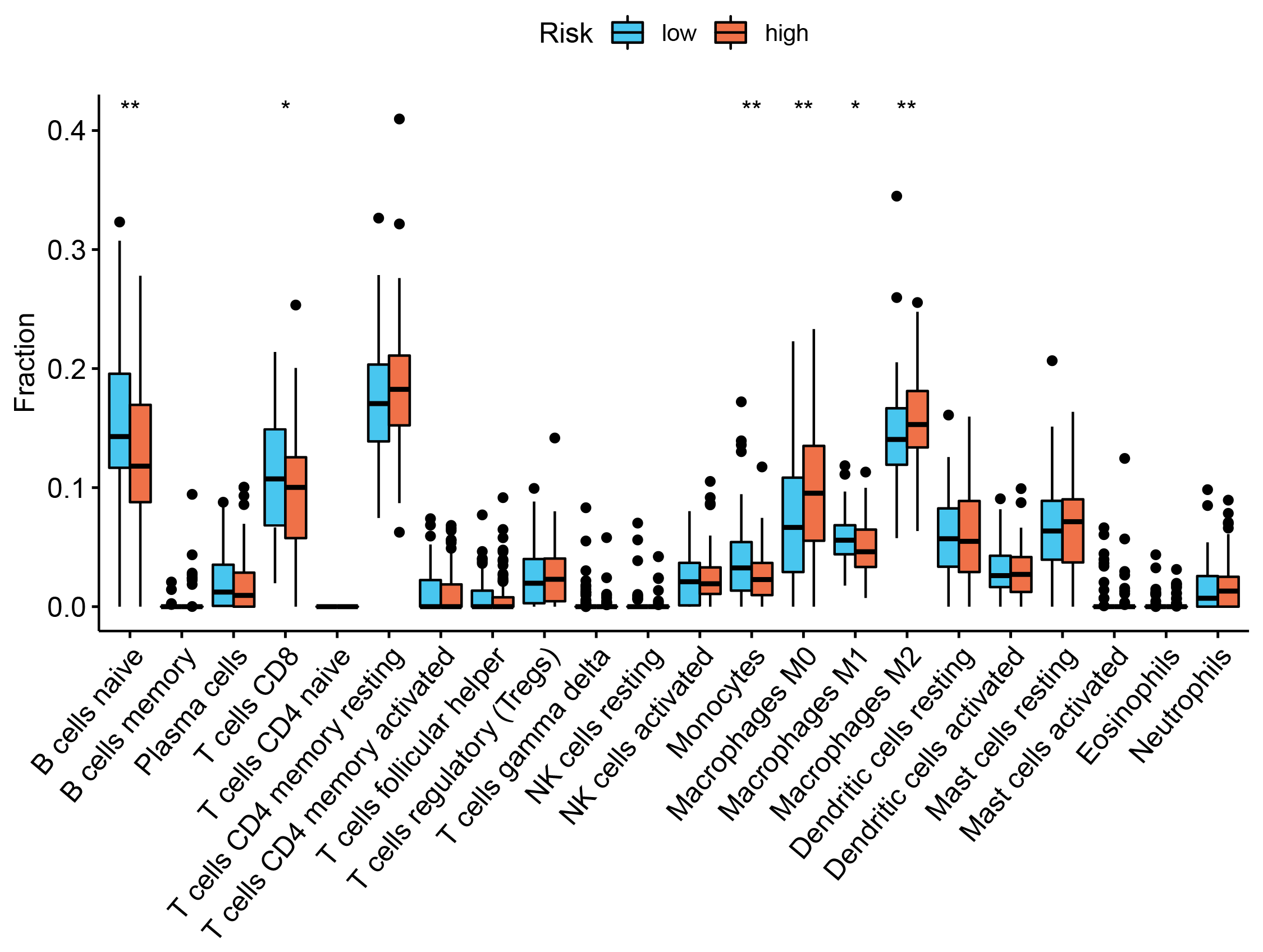

Supplement: Supplementary file 1 [file Image3.TIF]

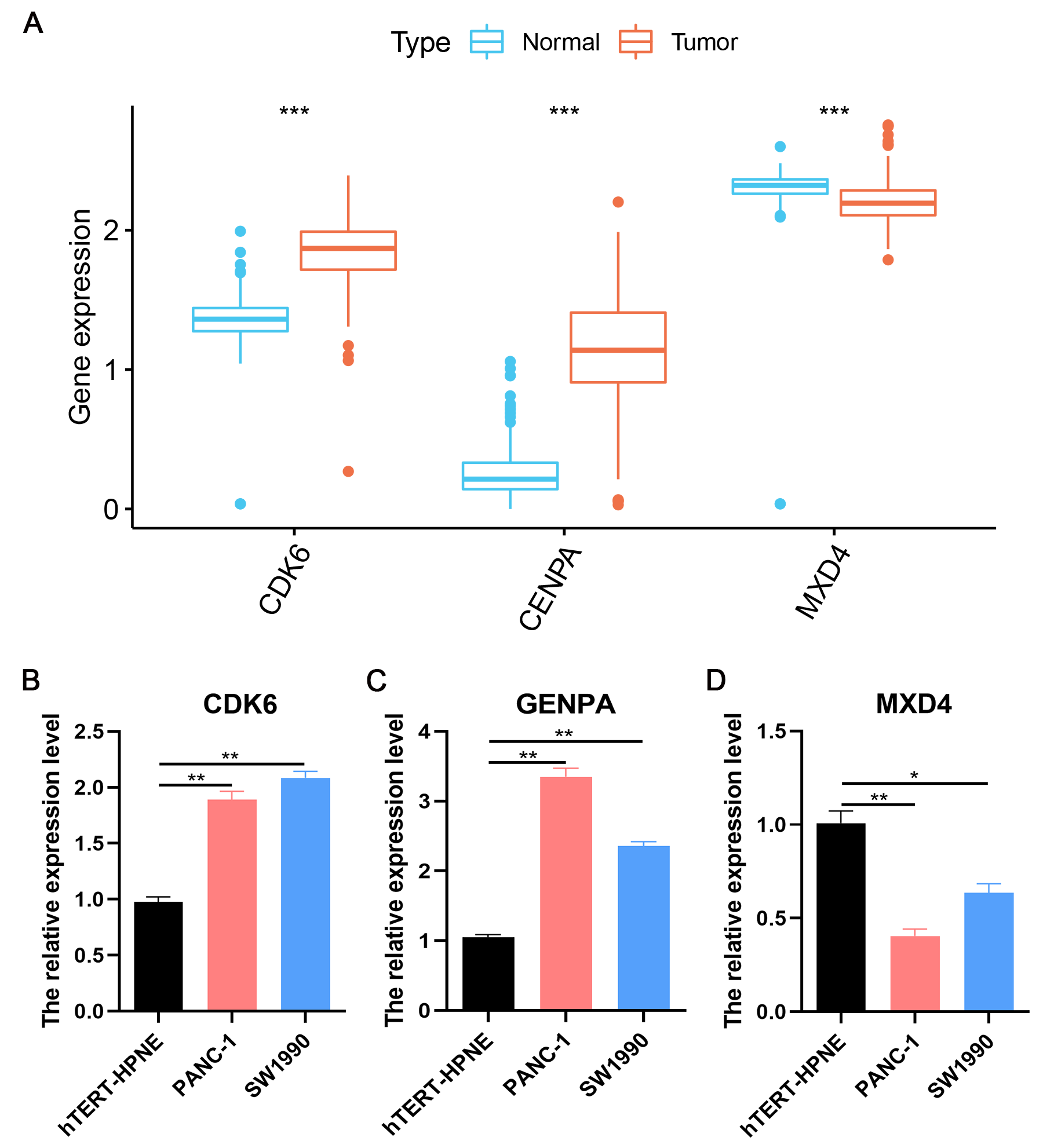

Supplement: Supplementary file 2 [file Image4.TIF]

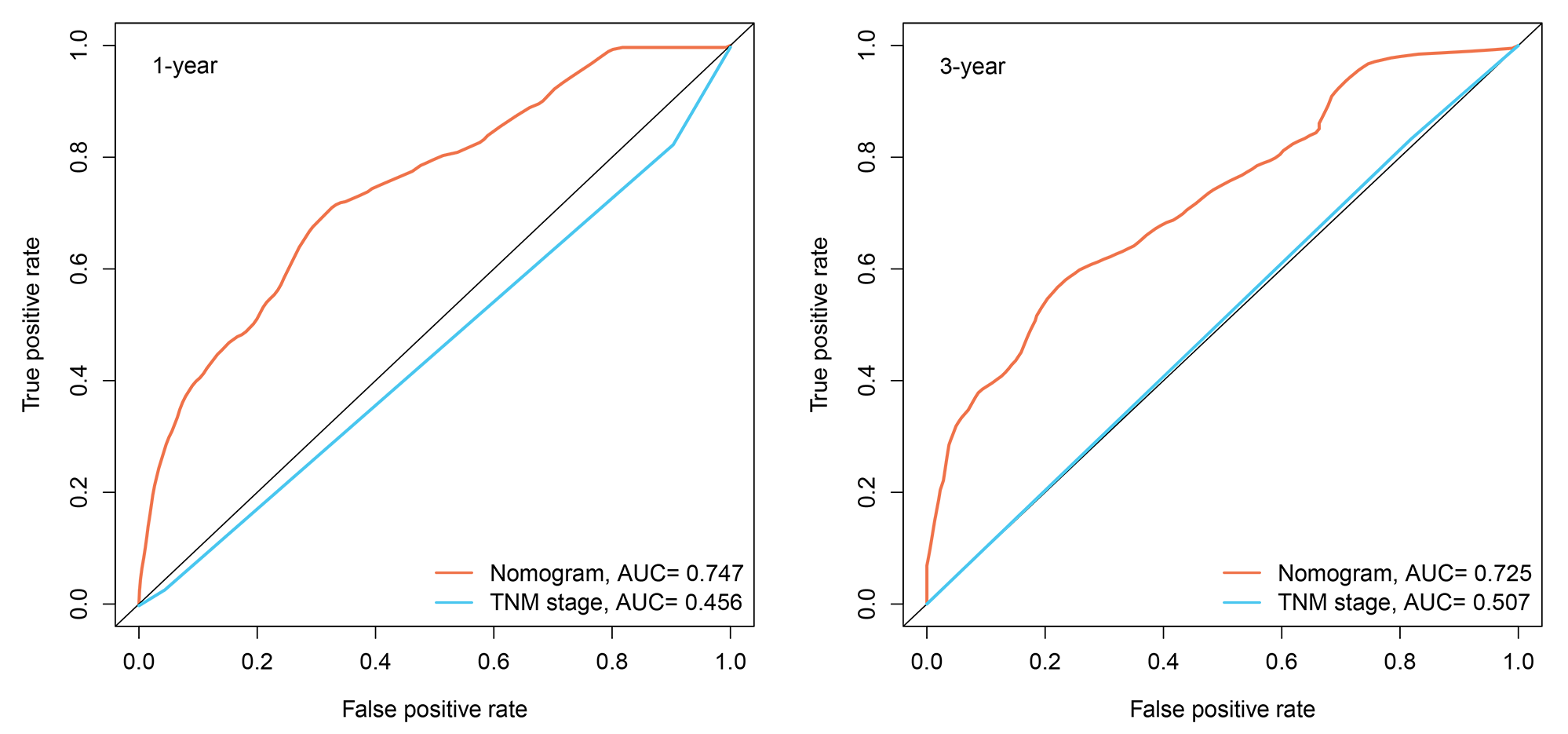

Supplement: Supplementary file 3 [file Image2.TIF]

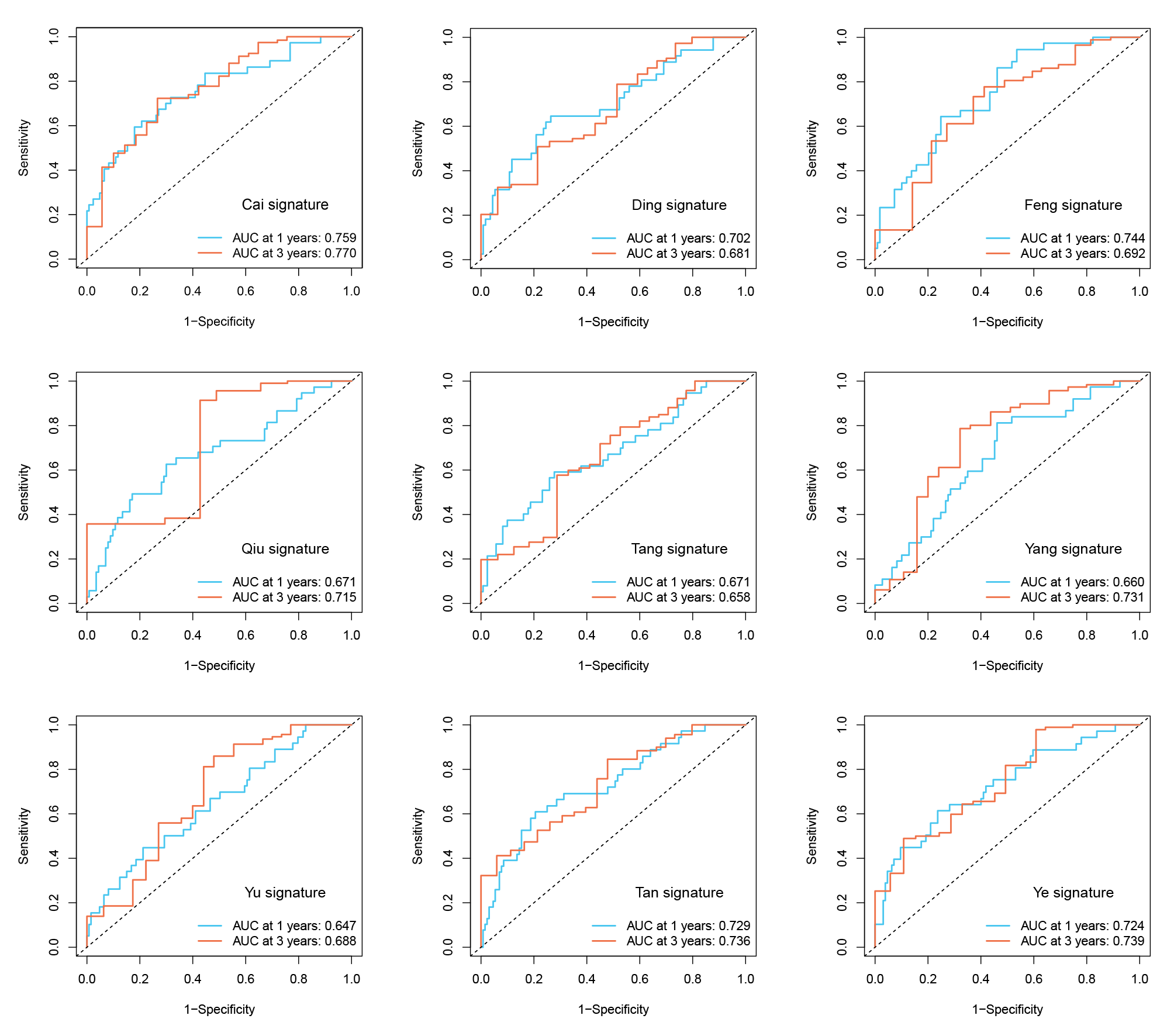

Supplement: Supplementary file 4 [file Image1.TIF]
